# The Perception of Instability and Legitimacy of Status Differences Enhances the Infrahumanization Bias among High Status Groups

**DOI:** 10.5964/ejop.v15i2.1585

**Published:** 2019-06-07

**Authors:** Cristina Mosso, Silvia Russo

**Affiliations:** a Department of Psychology, University of Torino, Torino, Italy; Webster University Geneva, Geneva, Switzerland; London School of Economics, London, United Kingdom

**Keywords:** infrahumanization, high-status group, status stability, status legitimacy, intergroup-relations

## Abstract

Previous research within the social identity framework has shown that perceptions of legitimacy and stability of status differences interactively determine cognitive, emotional, and behavioural responses to intergroup contexts. Whether such perceptions affect subtle forms of prejudice, namely infrahumanisation, is unknown. We examined if the perceptions regarding high status stability and legitimacy are associated to the infrahumanisation bias. We hypothesized that participants perceiving status differences as unstable and legitimate would report higher levels of infrahumanization than those who perceive status differences as stable and/or illegitimate. Participants (N = 439 Italian students enrolled in psychology courses) completed a structured paper-and-pencil questionnaire. We found that participants tended to attribute more negative secondary emotions to their ingroup (Italians) than to the outgroup (immigrants from Africa), indicating the presence of an infrahumanization bias. The results of a moderated regression aimed at predicting infrahumanization showed that high-status group members who perceived status differences as legitimate and unstable reported higher levels of infrahumanization than their counterparts did. The results attest the important and independent role of the perceptions related to the status for the debate on intergroup relations.

In this article, we ask whether subtle cues conveying the intergroup emotions are affected by the perception of social structure. This aim is relevant as in prior work on the intergroup relations the role of status and legitimacy is still controversial. Recent research has explored how the intergroup bias may be revealed through the infrahumanization, the tendency to attribute more humanity to the ingroup than to the outgroup. Unlike primary emotions, secondary emotions involve complex cognitive processes and are perceived to be uniquely human. For example, we can easily attribute sadness (primary emotion) to both humans and animals, while we would attribute disappointment (secondary emotion) to humans only. As such, the infrahumanization bias reflects the differential attribution of secondary emotions to ingroup vs. outgroup members. Several studies have shown that this bias characterizes a variety of intergroup and interpersonal relationships, ranging from ethnic to work to quasi-minimal groups (for a review see Demoulin et al., 2009; Leyens, 2009; Vaes, Leyens, Paladino, & Miranda, 2012), and that it is related to both animalistic and mechanistic dehumanization (Martínez, Rodriguez-Bailon, Moya, & Vaes, 2017).

The infrahumanization bias is very pervasive and recent studies showed that it is independent from the status-relationship between groups. For example, Leyens et al. (2001) found that Canarians (low-status group) tended to attribute fewer secondary emotions to Spanish people (high-status group) to the same extent as Spanish people did. Similarly, Rodriguez-Perez, Delgado-Rodriguez, Betancor-Rodriguez, Leyens, and Vaes (2011) examined the infrahumanization of outgroup members throughout the world in relation to similarity, intergroup friendship, knowledge of the outgroup, and status. Among these factors, status was the only one that was not related to outgroup dehumanization (for similar results see also Demoulin et al., 2005; Paladino et al., 2002; Paladino & Vaes, 2009). Altogether these results show that infrahumanization is not a prerogative of high-status groups, leading researchers to conclude that intergroup status differences are conditions neither necessary nor sufficient for infrahumanization to occur (Leyens, 2009).

However, less in known about whether perceptions of status differences affect expressions of the infrahumanization bias. Many studies reported that perceptions of legitimacy and stability of status differences play a fundamental role in explaining intergroup dynamics: They interactively determine the cognitive, emotional, and behavioural responses to the intergroup context (see Bettencourt, Dorr, Charlton, & Hume, 2001 for a comprehensive meta-analysis on this topic). In this study, we suggest that the perceptions of status differences might affect the infrahumanization bias more than the status-relationship between groups itself.

In pioneering work conducted within the social identity theory framework, Turner and Brown (1978) reported that high-status group members showed greater ingroup favoritism than low-status groups when status differences were perceived as legitimate but unstable. According to Turner and Brown, high-status groups are more likely to feel insecure when status differences are legitimate but the hierarchy seems to be unstable because the instability of status hierarchy represents a threat to the high-status group’s positive social identity and, consequently, leads to a sense of insecurity. Subsequent research supports this idea by showing that, when people feel that their high status is justified, they will be more likely to react by increasing ingroup identification and ingroup favoritism (Capozza, Andrighetto, Di Bernardo, & Falvo, 2012). In addition, perceptions of instability can be threatening for high-status group members as they put at stake their dominant role in society and can consequently lead to group protection reactions (Johnson, Terry, & Louis, 2005). This argument has further been supported by Scheepers, Ellemers, and Sintemaartensdijk (2009) who showed that physiological reactions signalling increased threat occurred when high-status group members were told intergroup status differences were unstable or likely to change.

Despite the importance of stability and legitimacy perceptions in shaping intergroup dynamics, the relationships between these perceptions and the infrahumanization bias, a kind of emotional prejudice (Leyens et al., 2003), has been largely neglected (but see Russo & Mosso, 2019 for an exception).

## The Study

In the current research, we focused on the role played by the *perception* of stability and legitimacy of socio-economic status differences in enhancing/reducing expressions of infrahumanization bias. We examined expressions of infrahumanization bias among Italians by comparing primary and secondary emotions attributed to the Italians themselves (high-status, ingroup) and to immigrants from Africa (low status, outgroup). We expected the perception of instability to be positively related to infrahumanization, especially when status differences are perceived as legitimate. In other words, participants perceiving status differences as unstable and legitimate should report higher levels of infrahumanization than those who perceive status differences as illegitimate and stable (H1).

## Method

### Participants and Procedure

Four-hundred and twenty-nine students (76.6% women; *M_age_* = 23.16, *SD* = 3.16) enrolled in psychology courses at a University in a large Italian city gave their informed consent. Their participation was voluntary and anonymous. Participants completed a structured paper-and-pencil questionnaire, presented as part of a study on attitudes and opinions of European students. All data and study materials are available from the authors upon request.

### Measures

#### Perception of Status Instability and Legitimacy

We asked participants to think about immigrants from Africa and to indicate, on a 7-point scale, whether: 1) socio-economic status differences between immigrants from Africa and Italians can change in the future (1 = very unlikely, 7 = very likely); 2) socio-economic status differences between immigrants from Africa and Italians were legitimate (1 = very illegitimate, 7 = very legitimate).

#### Infrahumanization

Participants have been presented four scenarios (texts were translated and adapted from Brown, Eller, Leeds, & Stace, 2007) in which the protagonists had a positive or negative experience. An example of positive experience is “[Italian name/name of immigrant from Africa] plays volleyball and is part of the University team. In the last game of the season he scored the winning point against the rival team”; an example of negative experience is “[Italian name/name of immigrant from Africa] loves art and his parents are both artists. He thought he would have received an excellent judgement by the committee of a painting competition but he found out he received a very bad one.” The order of the presentation was randomized. Two of the protagonists were said to be Italian and the other two to be immigrants from Africa. After reading each scenario participants had to check, in a list of eight emotions, all the emotions that the protagonist may have felt after the episode described. Four were primary emotions (happy, pleased, sad, angry) and four were secondary emotions (proud, good-mood, disappointed, ashamed) (Martin, Bennett, & Murray, 2008).

#### Control Variables

Among socio-demographic variables, we considered gender (1 = male), age, and political orientation (7-point left-right axis).

## Results

We examined the infrahumanization measure by summing up primary and secondary emotions attributed to ingroup and outgroup members across the four scenarios. We then conducted a 2 (protagonist Group membership: Ingroup vs. Outgroup) X 2 (type of Emotion: Primary vs. Secondary) X 2 (Valence of emotion: Positive vs. Negative) within-participants ANOVA. All the direct effects and two-way interactions were significant, and they were further qualified by a significant three-way interaction between factors, *F*(1, 428) = 143.31, *p* < .001, $\eta_{p}^{2}$ = .25. In order to clarify this interaction, we ran two 2 (protagonist Group membership: Ingroup vs. Outgroup) X 2 (type of Emotion: Primary vs. Secondary) ANOVAs by considering positive and negative emotions separately. As concerns positive emotions, only the main effect of emotion was significant, *F*(1, 428) = 170.08, *p* < .001, $\eta_{p}^{2}$ = .28, with secondary emotions selected more often (*M* = 3.06) than primary emotions (*M* = 2.26). When analyzing negative emotions we found significant main effects for group and emotion, but these effects were qualified by a significant two-way interaction *F*(1, 428) = 251.15, *p* < .001, $\eta_{p}^{2}$ = .37. The ingroup-outgroup difference in emotions attribution was noticeably greater for secondary emotions (*M_ingroup_* = 1.27, *M_outgroup_* = 0.26), *t*(428) = 32.06, *p* < .001, *d* = 4.07, than for primary emotions (*M_ingroup_* = 1.15, *M_outgroup_* = 1.00), *t*(428) = 3.87, *p* < .001, *d* = 0.49. We computed an index of infrahumanization by subtracting secondary negative emotions attributed to outgroup members from secondary emotions attributed to ingroup members (cf. Brown et al., 2007).

**Figure 1:**
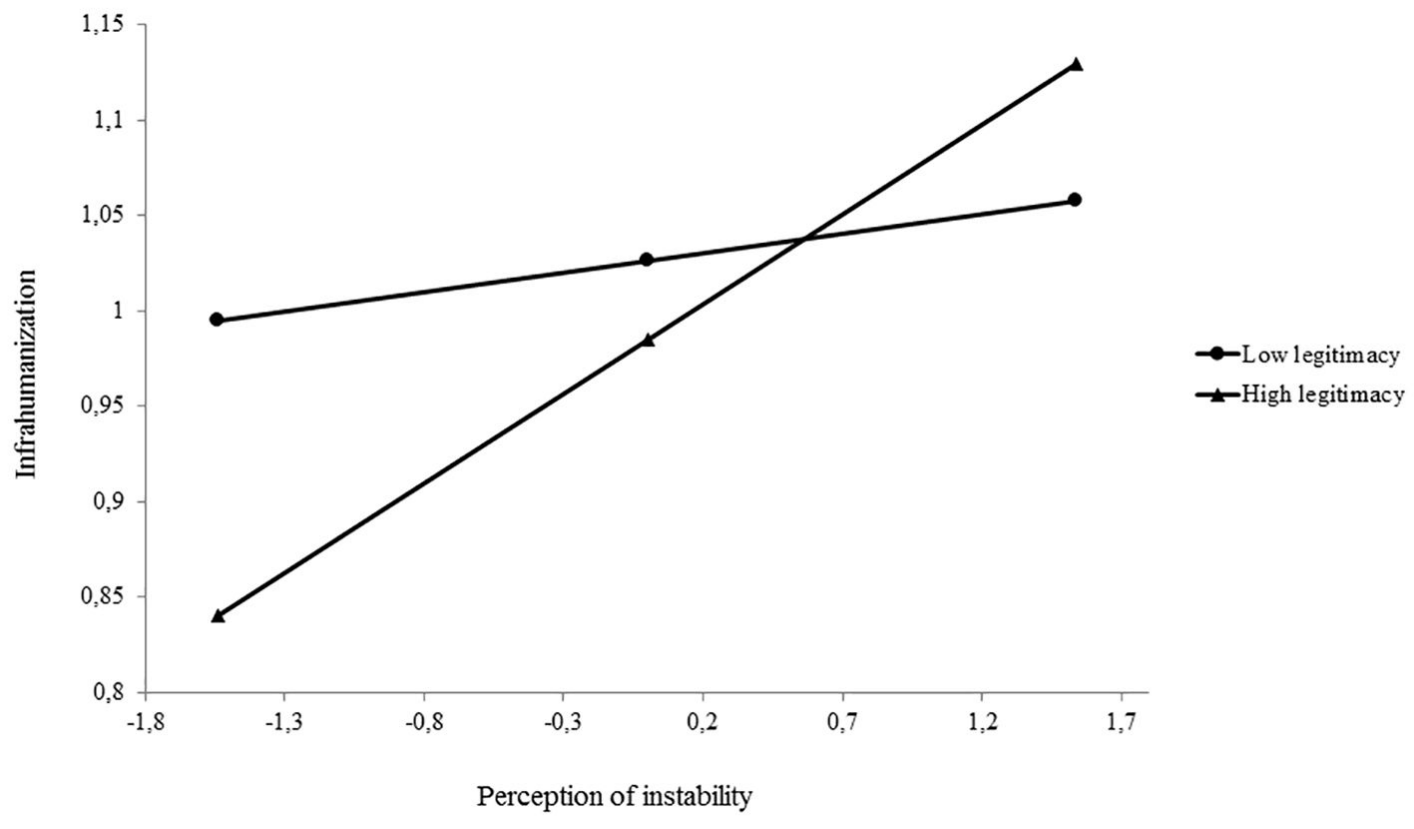
Moderating effect of the perception of status legitimacy on the relationship between the perception of instability and infrahumanization.

To test the hypothesis advanced, we ran a moderated regression aimed at predicting infrahumanization. As shown in Table 1, the perception of instability was significantly and positively related to the dependent variable. Adding the interaction between perception of legitimacy and instability of status differences (both mean centered), the explained variance of the model significantly increased, Δ*F*(1, 394) = 3.88, *p* < .05 (*R*^2^ = 07). The simple slopes analysis with perception of instability as predictor and perception of legitimacy as moderator showed that, in line with our expectations, the perception of instability had a positive and significant effect on infrahumanization among participants who perceived status differences as legitimate (+1 *SD*), *simple slope* = .09, *t*(394) = 3.34, *p* < .001, but not among those who perceived status differences as illegitimate (-1 *SD*), *simple slope* = .02, *t*(394) = .73, *p* = .47 (cf. Figure 1).

**Table 1:** Moderated Regression Analysis Predicting Infrahumanization

Predictor	Infrahumanization			
	*B*	*SE*	*t*	β
Intercept	0.92***	0.04	23.73	
Gender	-0.16***	0.04	-4.21	-0.21
Age	-0.01	0.01	-0.98	-0.05
Political orientation	0.02	0.02	0.63	0.03
Perception of legitimacy	-0.02	0.03	-0.55	-0.03
Perception of instability	0.06**	0.02	2.75	0.14
Legitimacy X Instability	0.03*	0.02	1.97	0.10

*p < .05. **p < .01. ***p < .001.

## Discussion

The present research investigated the relationship between high-status group members’ perceptions of instability and legitimacy of their own superiority and infrahumanization. Social identity research has shown that high-status group members tend to show high levels of ingroup favouritism and prejudice toward the outgroup when they perceive status differences as legitimate and unstable (Turner & Brown, 1978). Somewhat surprisingly, recent studies on the infrahumanization bias have shown that this form of subtle prejudice does not change as a function of social status (e.g., Leyens et al., 2001). However, the role played by the perception of sociostructural variables at an explicit level of analysis has never been explored; we aimed at testing the role of perceived stability and legitimacy among high-status group members in enhancing/reducing the infrahumanization bias.

Our findings, in line with Leyens et al. (2001), indicated that participants tended to attribute more negative secondary emotions to their ingroup (Italians) than to the outgroup (immigrants from Africa). For example, they tended to select more often “disappointment” to describe the feelings of an Italian person in a negative situation, than to describe the feelings of an immigrant. Given that secondary emotions are attributed uniquely to humans (and not to animals), this differential attribution has been described as a form of humanity denial.

Interestingly, in line with Turner and Brown (1978), we also found that the perception of the status differences as unstable and legitimate had a positive impact on the infrahumanization bias. In other words, participants who thought that it was right and fair for them to have a higher status compared to immigrants and, at the same time, perceived that such high position at risk (likely to change for worst) showed a tendency to perceive immigrants as less human (i.e., attributing less secondary emotions to immigrants than to Italians). This result has two main theoretical implications. The first implication is related to the role of status differences in shaping expressions of infrahumanization bias. Even though previous studies showed that this bias was not related to group hierarchies, the approach of focusing on ethnic, national or regional groups has been questioned in that these status differences may be perceived differently as concerns their extent and legitimacy (Vaes et al., 2012). Our findings support this argument by showing that the perception of legitimacy and stability of status differences matters for the manifestation of the infrahumanization bias.

The second one is related to the role of perceived threat to one’s own dominant status position in the society. On the one hand, theorists have linked the perception of threat to prejudice toward outgroups, and toward immigrants in particular (e.g., Stephan & Stephan, 1996), concluding that there is systematic evidence that high levels of perceived threat are strongly associated with negative attitudes toward outgroups (Riek, Mania, & Gaertner, 2006). On the other hand, research showed that the perception of instability of status differences is threatening for high status group members (e.g., Scheepers et al., 2009). The results gained in the current study indirectly supported the idea that perception of instability of one’s own superiority can foster the belief that one’s ingroup is more human than the outgroup because of increased threat perception. Importantly, this study expanded on previous research by showing that the potential effect of threat also applies to more subtle forms of prejudice (i.e., infrahumanization) than the ones traditionally considered, which today are socially disapproved and normatively sanctioned (Dovidio & Gaertner, 2004).

This study contributed to a deeper understanding of the relationships between infrahumanisation and the perception of socio-structural variables; notwithstanding, we have to acknowledge some limitations. First, our focus was limited to high-status group members. Future studies should focus on low-status groups as well, as different patterns of associations between perceived stability/legitimacy and infrahumanization could emerge. For example, research within the social identity theory suggests that, when status differences are perceived as legitimate, members of low status groups show outgroup favoritism (e.g., Turner, 1980). Second, we assessed the perception of stability and legitimacy through two single-item measures. Even if similar single-items have been used as measures of stability and legitimacy in previous research (e.g., Jost, Kivetz, Rubini, Guermandi, & Mosso, 2005), including the pioneering work by Turner and Brown (1978), future studies adopting more reliable measures (see Federico, 1999 for an example of a four-items scale to measure perception of legitimacy of status differences) will be welcome. Third, we found the infrahumanization effect for negative emotions only, which may be due to the limited number of emotions available in our dataset. Future studies should examine a larger number of positive and negative emotions.

### Conclusion

Research within the social identity theory framework emphasizes the importance of the perception of status differences (mainly legitimacy and stability) in shaping intergroup bias. However, the relationship between these perceptions are associated to the infrahumanization bias—is controversial. We filled this gap by showing that high-status group members who perceived status differences as legitimate and unstable reported higher levels of infrahumanization compared to their counterparts.

In times of increasing migration flows, we witness everyday discriminatory acts and prejudiced attitudes towards the newcomers. The findings from this study indicate that the specific perceptions that natives hold about their alleged status superiority do matter in expressions of infrahumanization, in particular the perception of legitimacy of their own position. The nature of sociostructural characteristics of intergroup relations should then inform present-day debates on the ethnic diversity of our societies, and the political discourses should be free of superiority legitimizing rhetoric.
